# Design, Synthesis and Thermal Energy Storage Properties of Polyurethane-Based Solid–Solid Phase Change Materials Using Trihydroxy Compounds as Chain Extenders

**DOI:** 10.3390/molecules31091426

**Published:** 2026-04-26

**Authors:** Ting Zhang, Yuxin Zhang, Lan Li, Xiaobing Lan, Changzhong Chen

**Affiliations:** Hunan Provincial Key Laboratory of Xiangnan Rare-Precious Metals Compounds Research and Application, School of Chemistry and Environmental Science, Xiangnan University, Chenzhou 423000, China; 18975714404@163.com (T.Z.); m13873250586@163.com (Y.Z.); 19807353687@163.com (L.L.)

**Keywords:** solid-solid phase change materials, crosslinked polyurethanes, polyethylene glycol, chain extender, thermal energy storage

## Abstract

Three crosslinked polyurethane copolymers were successfully synthesized as polymeric solid–solid phase change materials (SSPCMs) for thermal energy storage. These materials were fabricated utilizing trihydroxy compounds (glycerol, triethanolamine, and trimethylolethane) as chain extenders and polyethylene glycol (PEG) as the phase change functional segment. A comprehensive suite of characterization techniques was employed to investigate the chemical structures, thermal properties, and crystalline behaviors of the resulting SSPCMs. Fourier transform infrared (FTIR) spectroscopy confirmed the successful synthesis of the crosslinked polyurethane network. Polarizing optical microscopy (POM) and wide-angle X-ray diffraction (WAXD) analyses revealed that all three SSPCMs exhibit regular spherulitic morphologies with sharp diffraction peaks resembling those of pure PEG, although variations in spherulite size and diffraction intensity were observed among the samples. Differential scanning calorimetry (DSC) demonstrated the reversible latent heat storage and release capabilities of the synthesized SSPCMs, with a maximum endothermic enthalpy (Δ*H_endo_*) of 115.7 J/g. Furthermore, thermal cycling tests and thermogravimetric (TG) analysis verified their exhibit excellent reusability, thermal reliability, and high thermal stability.

## 1. Introduction

Thermal energy storage, utilizing solar energy or industrial waste heat, serves as a viable alternative to fossil fuel-based heating and cooling systems. This approach contributes to the reduction of carbon dioxide emissions and alleviates the demand for expensive peak-load power and heat generation facilities [1,2,3,4,5]. As an efficiency and promising thermal energy storage (TES) technique, latent heat storage based on phase change materials (PCMs) exhibits broad application prospects in numerous fields, including intelligent building climate control, temperature regulation systems, thermoregulatory textiles, agricultural greenhouses, etc. [6,7,8,9,10,11]. PCMs are capable of absorbing and releasing considerable latent heat within a narrow temperature range during the reversible melting and solidification processes.

Polyethylene glycol (PEG) has been extensively researched and applied as a classic solid–liquid PCM over the past few decades due to its large heat storage capability, wide range of phase change temperatures, low vapor pressure in the molten state, good thermal and chemical stability, non-toxicity, and non-corrosiveness [12,13,14,15,16,17,18]. However, Pure PEG suffers from serious liquid leakage upon melting. Consequently, this inherent drawback necessitates encapsulation to prevent the leakage of molted phase, which greatly restricts its practice applications in TES. To avoid the leakage of molted PEG without relying on special containers or packaging, shape stabilization using inorganic or polymer materials is usually adopted [19,20,21]. Essentially, the phase transition of PEG in these shape-stabilized (or form-stable) composite PCMs, which have difficulty maintaining good thermal reliability after repeated cycles.

Recently, PEG-based polyurethane (PU) copolymers featuring solid–solid phase transition characteristics have garnered increasing attention due to their unique advantages, such as the absence of liquid generation, minimal volume changes during phase transition, enhanced design flexibility, and extended lifespan [22,23,24,25,26]. In the molecular architecture of PEG-based PUs, PEG serves as the phase-change functional segment (soft segment), and is covalently bonded to diisocyanates (hard segment), ensuring that the material remains in a solid state even when the surrounding temperature is above the melting point of pure PEG.

Various PEG-based copolymers have been synthesized and characterized as polymeric SSPCMs for energy storage applications. In these systems, polymers with multiple hydroxyl groups (e.g., cellulose [27,28], cellulose diacetate [29], poly (vinyl alcohol) [30]) or small molecular compounds with multiple hydroxyl groups (e.g., Span 80 and Tween 80 [31], caster oil [32], 1,4-butanediol [33,34], pentaerythritol [24], sorbitol [35], glucose [36], β-cyclodextrin [37], Boltorn^®^ H20 [23,38], N-methyldiethanolamine [39]) are frequently employed as the molecular skeleton or chain extenders. In the synthesis of PEG-based PUs, the primary functions of the chain extender are to increase the length of the hard segment and the overall molecular weight of the copolymers. Consequently, as an integral component of the hard segment, the chain extender significantly influences the phase change properties of the SSPCMs. While Pielichowska et al. [34] investigated the influence of the chain extender BDO on properties of PEG-MDI-BDO PCMs, systematic discussions regarding the impact of different chain extenders on thermal properties and energy storage capabilities remain scarce in the literature.

In this work, the trihydroxyl compounds, glycerol (Gl), triethanolamine (TEA) and trimethylolethane (TME) were individually employed as chain extenders. Using diphenylmethane-4,4′-diisocyanate (MDI) serving as the crosslinker, a series of novel PEG-based PU copolymers serving as polymeric SSPCMs were synthesized via crosslinking copolymerization, maintaining an identical reactant mass ratio. We systematically compare three PEG-based crosslinked PU SSPCMs utilizing Gl, TEA and TME as trihydroxy chain extenders to elucidate the effect of the chain extender structure on the crystalline, phase change and thermal properties of the SSPCMs. Comprehensive characterization, including FTIR spectroscopy, polarizing optical microscopy (POM), wide-angle X-ray diffraction (WAXD), differential scanning calorimetry (DSC) and thermogravimetry (TG) analysis, was conducted to analyze the chemical structure, crystalline properties, phase transition behavior, and thermal stability of the synthesized SSPCMs.

## 2. Results and Discussion

### 2.1. FTIR Study

**Figure 1:**
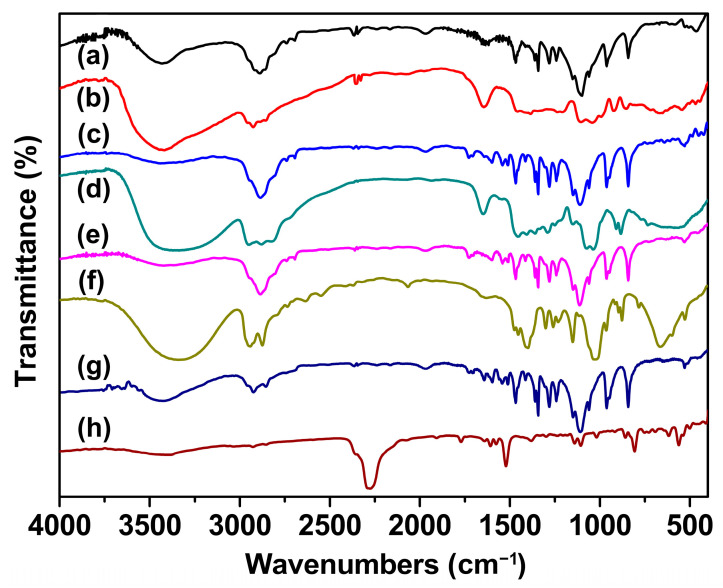
FTIR spectra of reactants and SSPCMs: (a) PEG; (b) glycerol; (c) SSPCM-1; (d) TEA; (e) SSPCM-2; (f) TME; (g) SSPCM-3; (h) MDI.

The FTIR spectra of the raw materials and synthesized SSPCMs are presented in Figure 1, with detailed peak assignments summarized in Table 1. As shown in Figure 1 (a) and Table 1, pure PEG exhibits two characteristic absorption bands associated with oxygen-containing functional groups: a broad peak at 3432 cm^−1^ attributed to O–H stretching vibration and peaks at 1242 cm^−1^ and 1100 cm^−1^ corresponding to C–O stretching vibration [12,13]. Additionally, C–H stretching and bending vibrations of PEG are observed at 2889 cm^−1^, 1467 cm^−1^, 1342 cm^−1^ and 842 cm^−1^. For the three trihydroxy chain extenders, broad and intense absorption bands due to O–H stretching vibration are detected at 3421 cm^−1^ for Gl (Figure 1 (b)), 3338 cm^−1^ for TEA (Figure 1 (d)) and 3342 cm^−1^ for TME (Figure 1 (f)). Meanwhile, MDI displays a sharp characteristic peak at 2276 cm^−1^, which is assigned to the –NCO stretching vibration (Figure 1 (h)). In the FTIR spectra of all synthesized SSPCMs (Figure 1 (c,e,g)), the C–O stretching peak and C–H stretching/bending vibrational peaks remain clearly visible, while the characteristic –NCO peak of MDI at 2276 cm^−1^ completely disappears. Moreover, the absorption band in the 3300–3500 cm^−1^ region becomes broader and less intense compared to those of PEG and the trihydroxy monomers. These observations indicate that the –NCO groups from MDI and the –OH groups from the reactants have been fully consumed during the crosslinking reaction. Furthermore, new characteristic peaks emerge at 1541 cm^−1^, 1727 cm^−1^ and 1599 cm^−1^, which are assigned to N–H bending (amide II band), C=O stretching of the urethane linkage (–NHCOO–), and aromatic C=C stretching of the benzene ring, respectively [35,36,37]. Based on the FTIR analysis, it is confirmed that the crosslinked structured polymeric SSPCMs have been successfully synthesized.

### 2.2. Crystalline Properties of SSPCMs

**Figure 2:**
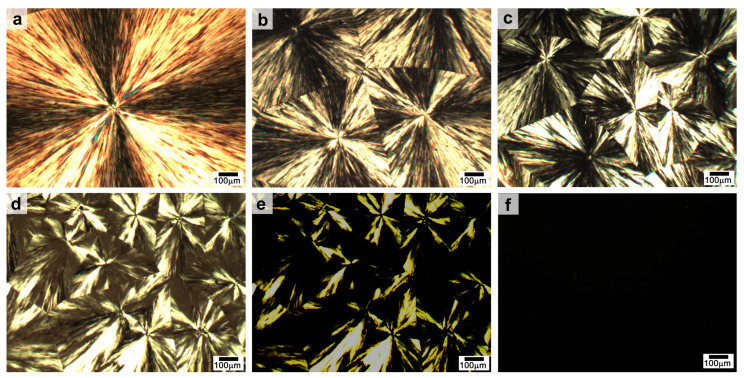
POM images: (**a**) PEG; (**b**) SSPCM-1; (**c**) SSPCM-2; (**d**) SSPCM-3; (**e**) SSPCM-3 at 55 °C; and (**f**) SSPCM-3 at 60 °C.

The crystallization morphology of PEG and the synthesized SSPCMs was investigated using a polarizing optical microscope (POM), as shown in Figure 2. The POM images of PEG (Figure 2a) reveals the formation of typical spherulites with a characteristic cross-petal shape. Similar spherulitic morphologies are also observed for all three SSPCMs (Figure 2b–d). PEG is a linear polymer with a highly symmetric and non-branched structure, which allows its flexible chains to readily organize into well-ordered crystalline domains [34]. Since no spherulites were observed for the three trihydroxyl chain extenders alone, it can be concluded that the spherulitic morphology in the SSPCMs originates from the crystallization of the (CH_2_CH_2_O)_n_ units within PEG segments.

Notably, the spherulite size of the SSPCMs is significantly smaller than that of pristine PEG. This morphological change suggests that a greater number of nucleation sites are generated during the crystallization of SSPCMs, which restricts the subsequent growth of the PEG spherulites. This observation aligns with the DSC and XRD analyses, which indicate that the overall crystallinity of the SSPCMs is reduced compared to that of pure PEG. This reduction is attributed to the restricted mobility of the PEG segments, a consequence of the crosslinked network structure and the incorporation of rigid, benzene ring-containing hard segments [24]. Furthermore, the spherulite size varies among the three SSPCMs, following the order of SSPCM-1 > SSPCM-2 > SSPCM-3. This trend reflects the distinct influence of each trihydroxy chain extender on the crystallization behavior of the PEG segments.

Upon heating from room temperature to just below the phase transition temperatures of SSPCMs, no significant changes in their spherulitic morphology were observed. When the temperature reached the phase transition points, some spherulites gradually disappeared from the field of view of the polarizing microscope (Figure 2e). As the temperature exceeded their respective phase transition points, the spherulites completely vanished under polarized light, leaving only darkness in the field of view (Figure 2f). Concurrently, no liquid flow was observed for any of the synthesized materials under a normal light microscope. These results confirm that the synthesized materials undergo a reversible phase transition from a crystalline state to an amorphous state, which is characteristic of a solid–solid phase change.

The observed solid–solid phase transition mechanism can be attributed to the unique microphase separation structure of the SSPCMs. The PEG segments serve as the soft, crystallizable domains responsible for latent heat storage, while the crosslinked network or rigid hard segments act as the structural skeleton. Upon heating, the thermal energy disrupts the ordered crystalline lattice of the PEG segments, causing them to transition into a disordered, amorphous state. However, the covalent bonds within the network prevent macroscopic flow, thereby maintaining the solid integrity of the material. This transition is characterized by a change in optical properties from birefringent (crystalline) to isotropic (amorphous), which is visually manifested as the disappearance of spherulites in POM.

**Figure 3:**
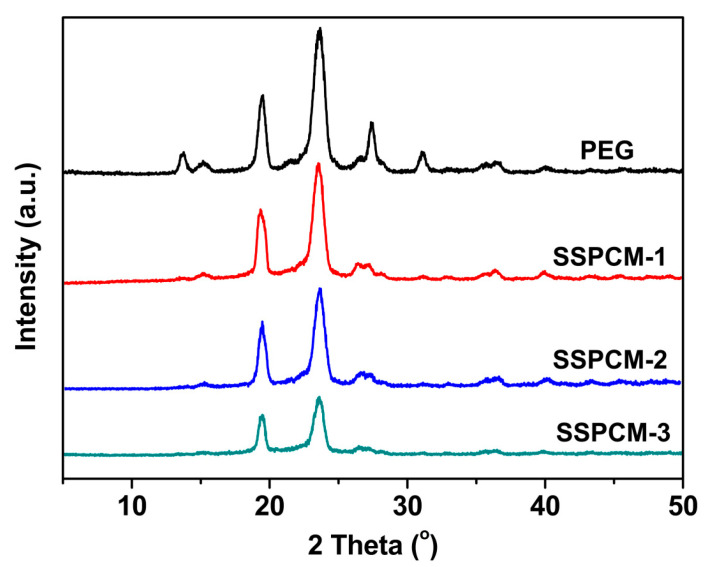
XRD patterns of PEG and SSPCMs.

The WAXD patterns of PEG and the SSPCMs are presented in Figure 3 to further elucidate their crystalline properties. Distinctly, two sharp diffraction peaks located at approximately 19.3 and 24.6 are observed in all WAXD curves, indicating that the synthesized SSPCMs retain the same crystal unit cell structure as pristine PEG [24,25,30]. However, the diffraction intensity of the SSPCMs is significantly lower than that of pure PEG, revealing a reduction in overall crystallinity. This suggests that the crosslinking network structures of SSPCMs impose substantial constraints on the mobility of the PEG segments, thereby hindering their ability to form well-ordered crystalline domains. Furthermore, the diffraction intensity of the SSPCMs follows the order SSPCM-1 > SSPCM-2 > SSPCM-3, a trend that is in excellent agreement with the spherulitic morphologies observed in the POM analysis. This variation is likely attributed to the different steric hindrance effects of specific chain extenders used, which modulate the segmental mobility of the PEG chains to varying degrees.

### 2.3. Phase Change Properties of SSPCMs

**Figure 4:**
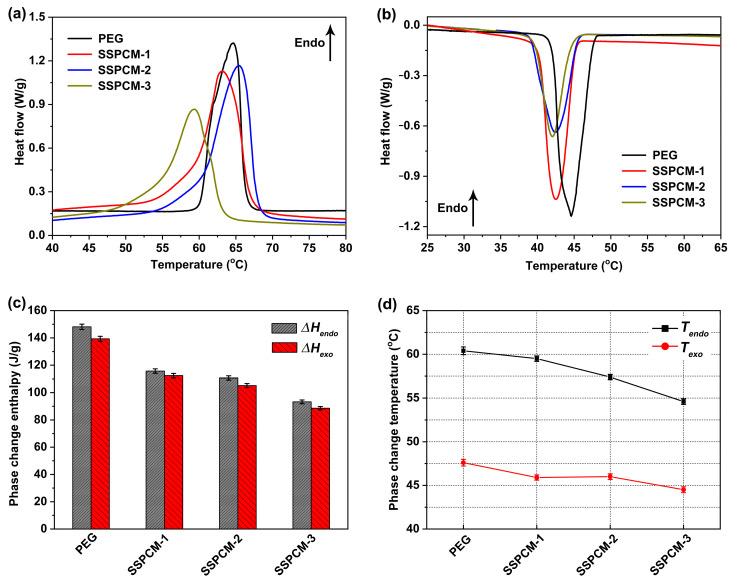
(**a**) DSC curves in endothermic process; (**b**) DSC curves in exothermic process; (**c**) phase change enthalpies; (**d**) phase change temperatures of pure PEG and the SSPCMs.

Phase change energy storage properties are the primary indicator for evaluating the potential of the synthesized materials in TES applications. Figure 4a,b present the DSC curves of PEG and the synthesized SSPCMs, and their detailed phase change property data are summarized in Figure 4c,d and Table 2. As shown in Figure 4a,b, distinct endothermic and exothermic peaks are observed for both PEG and the three SSPCMs below 100 °C, demonstrating their reversible latent heat storage and release capabilities. Thermal energy was stored and released via the solid–liquid phase transition of PEG and the solid–solid phase transition of the SSPCMs during heating and cooling cycles. Notably, the peak intensities in DSC curves of the SSPCMs are lower than those of pure PEG. As quantified in Figure 4c, the endothermic and exothermic enthalpies (Δ*H_endo_* and Δ*H_exo_*, corresponding to the enthalpies of fusion and solidification of PEG, respectively) of pure PEG are 148.1 J/g and 139.3 J/g, respectively. For the SSPCMs, these values range from 88 to 116 J/g, which is lower than those of pristine PEG. The slight but consistent discrepancy observed between the exothermic and endothermic enthalpies can be attributed to residual crystallization events occurring outside the defined integration limits of the exothermic peaks. Specifically, minor crystallization processes may occur prior to the formal onset of the main exothermic peak or persist beyond its conclusion. Although these events contribute to the overall enthalpy change, they are not fully captured within the integration bounds used for quantification. Consequently, this leads to a systematic underestimation of the total exothermic enthalpy, resulting in the observed deviation compared to the endothermic values.

Compared with pure PEG, the phase change energy storage density of SSPCMs is reduced to a certain extent. This is because both ends of the PEG segments in SSPCM molecules are strictly cross-linked by MDI, which restricts the directional arrangement of the PEG segments and prevents some of them from crystallizing. The WAXD patterns and POM images discussed above also confirm the deterioration of the crystalline properties of SSPCMs relative to PEG. According to previous studies [23,24,25,31,32,33,34,35,36,37,38], the phase transition enthalpies of PEG-based SSPCMs with a cross-linked PU structure generally decrease to some extent due to weakened crystallinity. However, SSPCM-1 stands out with a remarkably high enthalpy ratio (defined as the ratio of the endothermic enthalpy of SSPCM to that of PEG) [37] of approximately 78.11%. This value not only significantly surpasses those reported in most previous studies [25,27,28,29,30,33,35,36,37] but also demonstrates that SSPCM-1 retains exceptional energy storage capacity despite the constraints of the crosslinked network, highlighting its superior performance among PEG-based SSPCMs.

It is noteworthy that the phase change enthalpies of the three SSPCMs vary despite Gl, TEA and TME possessing the same number of hydroxyl groups for crosslinking. As shown in Figure 4c and Table 2, the Δ*H_endo_* or Δ*H_exo_* of the SSPCMs values follow the order SSPCM-1 > SSPCM-2 > SSPCM-3. The weight percentage of PEG segments and crystalline properties of SSPCMs are the dominant factors influencing the phase transition behaviors of SSPCMs [35]. Based on the reactant stoichiometry, the theoretical weight percentages of PEG segments in SSPCM-1, SSPCM-2 and SSPCM-3 are 93.44%, 93.02% and 93.23%, respectively, showing negligible differences. Consequently, the SSPCM exhibiting superior crystalline properties possesses higher phase change enthalpies. Furthermore, Figure 4d reveals that the endothermic/exothermic phase transition temperatures (*T_endo_*/*T_exo_*) of SSPCMs are slightly lower than those of PEG, which is likely attributed to the steric hindrance imposed by the crosslinking network on the PEG segments in SSPCMs [24]. The thermal transition of SSPCM-1, SSPCM-2 and SSPCM-3 spanned a range of approximately 10 °C, providing a broad operating window suitable for applications requiring gradual heat absorption.

### 2.4. Thermal Reliability and Stability of SSPCMs

**Figure 5:**
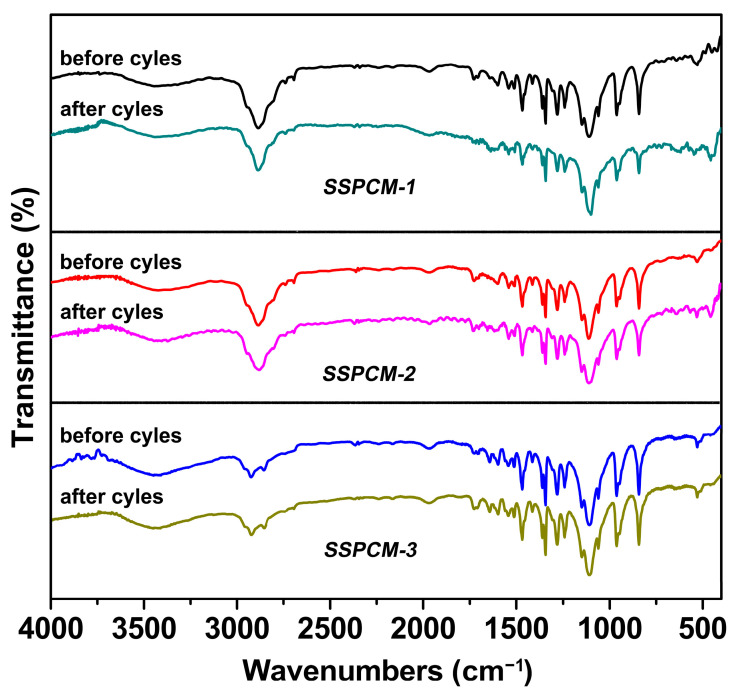
FTIR spectra of SSPCMs before and after the thermal cycling test.

Accelerated thermal cycling tests comprising 1000 heating–cooling cycles were conducted to evaluate characterize the thermal reliability of the SSPCMs. The mass loss of the three SSPCMs after thermal cycling was found to be negligible within the measurement uncertainty compared to the original samples. Figure 5 presents the FTIR spectra of the original and thermally cycled SSPCM-1, SSPCM-2 and SSPCM-3. Compared with the FTIR spectra of the original of samples, no new absorption peaks appeared, and all the original peaks remained at the same wavenumbers in the spectra of the thermally cycled samples. This indicates that the chemical structure of the SSPCMs remained unchanged and that no chemical degradation of SSPCMs occurred after the thermal cycles.

**Figure 6:**
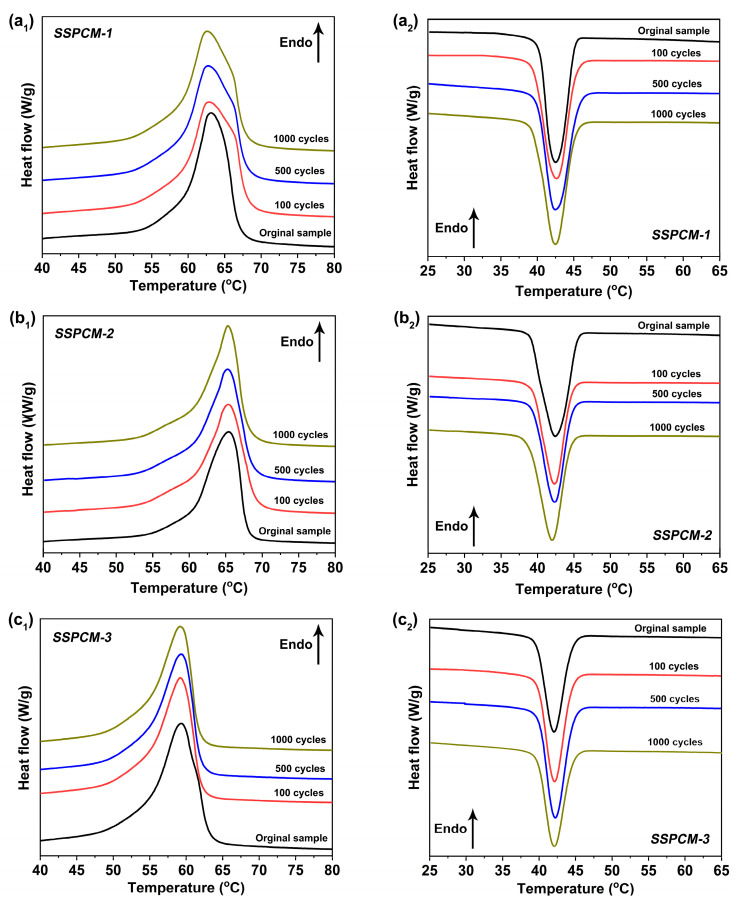
(**a_1_**–**c_1_**) are DSC curves in endothermic process; (**a_2_**–**c_2_**) are DSC curves in exothermic process for SSPCMs before and after different thermal cycles.

Figure 6 displays the DSC curves of the SSPCMs before and after varying numbers of thermal cycles. The phase change properties are summarized in detail in Table 2. The DSC curves for the three SSPCMs, in both endothermic and exothermic processes, after 100, 500, and 1000 cycles are nearly superimposable on those of the original samples, indicating remarkable consistency in their thermal behaviors. As detailed in Table 2, the phase change enthalpies and temperatures of the SSPCMs exhibit only negligible variations after thermal cycling compared to the original samples. For instance, the phase change enthalpy of SSPCM-1 decreased by less than 0.52% after 1000 cycles, while its phase change temperature remained stable within a 0.1 °C range. These minimal changes underscore the robust thermal stability of the SSPCMs. Collectively, the results from Figure 5 and Figure 6 and Table 2 confirm the excellent thermal reliability and repeatability of the SSPCMs, highlighting their strong potential for long-term TES applications.

**Figure 7:**
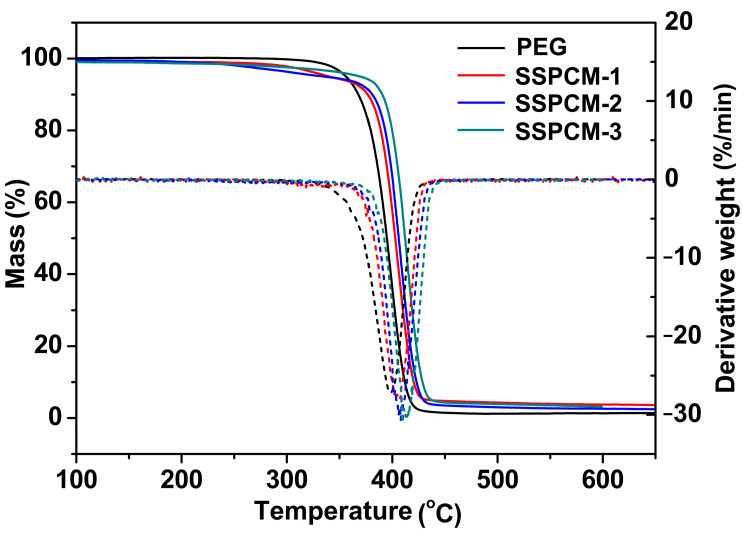
TG (the solid line) and DTG (the dot line) curves of PEG and SSPCMs.

To further investigate the thermal decomposition behaviors of SSPCMs, Figure 7 presents the TG and DTG curves of PEG and SSPCMs, with the detailed degradation data of all samples summarized in Table 3. Clearly, the pristine PEG power exhibits a mass loss of only about 0.10% at 100 °C and 3.50% at 350 °C, with its primary thermal degradation occurring in the temperature range of approximately 376.2 °C to 412.9 °C. It can be observed that the mass loss of the three SSPCMs is slightly higher than that of PEG at the same temperature, which may be attributed to the partial pyrolysis of the chain extender. Notably, the main thermal degradation of the three SSPCMs, corresponding to the decomposition of PEG segments, initiates at approximately 385 °C (SSPCM-1), 390 °C (SSPCM-2) and 396 °C (SSPCM-3), respectively. Polymer rupture and rearrangement reactions predominantly occur during this stage, contributing to the degradation of PEG segments and the subsequent formation of residual carbides. As shown in Table 3, the degradation temperatures (*T_onset_* and *T_end_*) of all the SSPCMs in this stage are higher than those of PEG, indicating that the crosslinked network structure of SSPCMs enhances their thermal stability. Moreover, the degradation temperatures of the SSPCMs exhibit a clear trend: SSPCM-1 < SSPCM-2 < SSPCM-3, which confirms that the PEG segments in SSPCM-3 are in the most tightly constrained state. This conclusion is further supported by the POM images and WAXD curves. In summary, the three synthesized SSPCMs demonstrate excellent thermal stability within their corresponding phase transition temperature ranges (below 100 °C), which is favorable for their potential application for thermal energy storage.

## 3. Materials and Methods

### 3.1. Materials

Glycerol (purity 99.5%), triethanolamine (purity 99%), and trimethylolethane (purity 98%) were obtained from Aladdin Chemistry Co., Ltd., Shanghai, China. PEG (average molecular weight 8000, purity 99%) was purchased from Macklin Biochemical Technology Co., Ltd., Shanghai, China and was dried in a vacuum oven at 100 °C for 6 h before use. Diphenylmethane-4,4′-diisocyanate (MDI, purity 99.8%) was purchased from Aladdin Chemistry Co., Ltd., China. The MDI was heated to 60 °C and kept in a vacuum oven for 2 h, then filtered by a heated filter. Anhydrous *N,N*-dimethylformamide (DMF, purity 99.8%) was provided by Aladdin Chemistry Co., Ltd., China) and used as received without further purification.

### 3.2. Synthesis of SSPCMs

**Figure 8:**
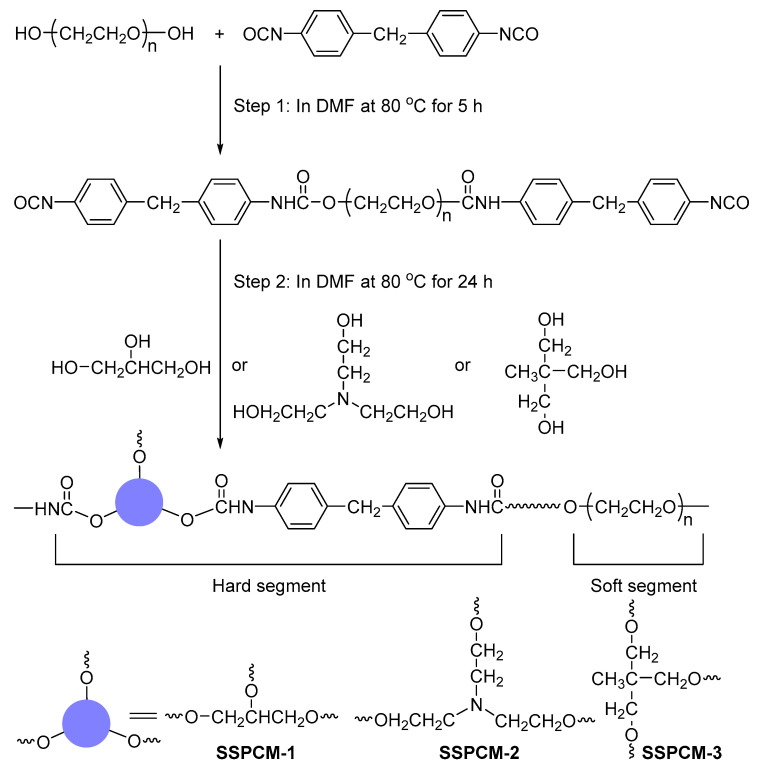
Schematic diagrams for preparing SSPCMs.

The synthesis of SSPCMs followed a two-step route, as depicted in Figure 8. In the first step, an anhydrous DMF solution containing PEG and MDI (in a 1:2 molar ratio) was prepared in a three-necked round-bottom flask equipped with a reflux condenser. The reaction proceeded under a nitrogen atmosphere at 80 °C for 5 h in a thermostatic oil bath [21,26]. In the second step, a DMF solution of Gl was added dropwise to the reaction mixture (PEG:Gl molar ratio = 3:2), and the reaction was continued for an additional 24 h under the same conditions. Subsequently, the mixture was poured into a beaker and cured in an oven at 80 °C for 24 h. To eliminate residual volatiles, the resulting product was dried in a vacuum oven at 40 °C for two weeks. The SSPCMs synthesized using TEA and TME as chain extenders were prepared following an identical procedure. The final products obtained with Gl, TEA, and TME were designated as SSPCM-1, SSPCM-2, and SSPCM-3, respectively.

### 3.3. Characterization

The FTIR spectra of the starting materials and the synthesized SSPCMs were recorded using a Fourier transform infrared spectrometer (NICOLET-760, Nicolet Co., USA) over the wavenumber range of 400–4000 cm^−1^. Prior to measurement, each sample was uniformly mixed with KBr powder and pressed into a transparent disk for analysis. The crystallization morphology of samples was examined using a polarizing optical microscope (XPN-300E, Changfang Optical Instrument Co., Ltd., Shanghai, China) equipped with a digital camera and a programmable hot stage. WAXD patterns of the samples were recorded using a wide-angle X-ray diffractometer (D8 Advance, Bruker-AXS, Germany) with Cu Kα radiation. Data were collected in the 2θ range of 5° to 50°, using a step size of 0.02° and a counting time of 0.1 s per step. The DSC and TG curves were performed using a simultaneous thermal analyzer (STA 449 F3 Jupiter^®^, Netzsch, Germany) under a nitrogen atmosphere. For DSC measurements, approximately 5–10 mg of each sample was sealed in an aluminum crucible and subjected to two heating–cooling thermal cycles between 25 °C and 100° C at a 2 °C/min heating/cooling rate. The DSC curve reported herein was derived from the second thermal cycle to eliminate the influence of the thermal history. TG analyses were conducted from 25 °C to 600 °C at a heating rate of 10 °C/min. The thermal reliability of the SSPCMs was evaluated through an accelerated thermal cycling test. Samples loaded in aluminum pans were subjected to 100, 500 and 1000 consecutive heating–cooling cycles between 25 °C and 100 °C on the hot stage. After the cycling tests, FTIR spectroscopy and DSC analysis of the cycled SSPCMs were measured under the same conditions as described above.

## 4. Conclusions

In summary, three PEG-based SSPCMs incorporating a polyurethane (PU) copolymer structure were successfully synthesized and characterized. Gl, TEA, and TME were employed as chain extenders to tailor the material properties. POM and WAXD analyses confirmed that the synthesized SSPCMs exhibit a typical solid–solid phase change mechanism characterized by a regular spherocrystalline morphology. The selection of the chain extender significantly influenced the crystallization and phase change properties of the resulting materials. Among the three, the SSPCM utilizing Gl as the chain extender (SSPCM-1) demonstrated the highest phase change enthalpies, with $\Delta H_{\mathrm{endo}}$ and $\Delta H_{\mathrm{exo}}$ values of 115.7 J/g and 112.4 J/g, respectively. Furthermore, comprehensive thermal cycling tests and TG analyses verified the excellent thermal reliability, cycling stability, and high thermal resistance of the SSPCMs. These results highlight the potential of the synthesized materials as promising candidates for efficient thermal energy storage applications.

## Figures and Tables

**Table 1:** The detailed compositions of the FTIR spectrum for the reactants and SSPCMs.

Characteristic Vibration	Characteristic Peaks (cm^−1^)			
	PEG	SSPCM-1	SSPCM-2	SSPCM-3
O-H stretching	3432	-	-	-
N-H stretching	-	3424	3424	3427
C-H stretch of CH_2_	2889	2886	2886	2926, 2856
Amide bond I	-	1727	1727	1727
C=C skeletal (benzene ring)	-	1599	1599	1599
Amide bond II	-	1541	1541	1541
C-H bending	1467, 1342	1467, 1343	1467, 1343	1467, 1343
C-O stretching	1242, 1100	1242, 1112	1242, 1113	1241, 1112
Crystallization band	963	963	963	963
Interim -CH_2_- group	842	842	842	842

**Table 2:** Phase change properties of SSPCMs before and after the thermal cycling test.

Sample		Endothermic Process			Exothermic Process		
		Δ*H_endo_* (J/g)	*T_endo_* (°C)	*T_peak_* (°C)	Δ*H_exo_* (J/g)	*T_exo_* (°C)	*T_peak_* (°C)
SSPCM-1	Original	115.7	59.5	63.2	112.4	45.9	42.5
	100 cycles	115.4	59.4	62.8	112.2	45.9	42.3
	500 cycles	115.2	59.4	62.8	112.2	46.0	42.3
	1000 cycles	115.1	59.4	62.6	111.9	45.9	42.2
SSPCM-2	Original	110.7	57.4	65.5	105.2	46.0	42.3
	100 cycles	110.7	57.3	65.0	105.2	46.0	42.2
	500 cycles	110.5	57.2	64.8	105.2	45.9	42.3
	1000 cycles	110.5	57.2	64.6	105.1	45.9	42.0
SSPCM-3	Original	93.3	54.6	59.2	88.6	44.5	41.9
	100 cycles	93.1	54.5	59.2	88.5	44.5	41.7
	500 cycles	93.0	54.6	59.3	88.5	44.4	41.7
	1000 cycles	93.0	54.6	59.3	88.5	44.4	41.4

**Table 3:** Degradation data from TG curves.

Samples	Degradation Interval (°C) *^a^*	Mass Loss (%)		
		At 100 °C	At 350 °C	At 600 °C
PEG	376.2–412.9	0.10	3.50	98.67
SSPCM-1	384.9–419.2	0.58	5.68	96.26
SSPCM-2	390.3–421.6	0.46	5.57	97.39
SSPCM-3	395.9–427.6	0.93	3.83	96.80

*^a^* The degradation interval is from *T_onset_* (the onset degradation temperature of TG curve) to *T_end_* (the end degradation temperature of the TG curve).

## Data Availability

Data are contained within the article.
